# Proliferation of Vascular Smooth Muscle Cells under ox-LDL Is Regulated by *Alismatis rhizoma* Decoction via InhibitingERK1/2 and miR-17∼92a Cluster Activation

**DOI:** 10.1155/2020/7275246

**Published:** 2020-08-22

**Authors:** Julian Shen, Wei Wei, Xialei Wang, Jingda Yang, Lu Lu, Xinru Lv, Xiehua Xue

**Affiliations:** ^1^The Affiliated Rehabilitation Hospital, Fujian University of Traditional Chinese Medicine, Fuzhou 350003, China; ^2^College of Rehabilitation Medicine, Fujian University of Traditional Chinese Medicine, Fuzhou 350112, China; ^3^Fujian Provincial Rehabilitation Industrial Institution, Fujian Provincial Key Laboratory of Rehabilitation Technology, Fuzhou, China

## Abstract

*Context*: *Alismatis rhizome* decoction (*AD*) exhibits antiatherosclerotic activities. The activity of *AD* against vascular smooth muscle cell (VSMC) proliferation remains unclear. *Objective*. The mechanisms and effects of *AD* on oxidized low-density lipoprotein (ox-LDL)-induced VSMC proliferation were explored. *Materials and methods*. The male SD rats were fed with *AD* (2.56 g/mL) or 0.9% NaCl by oral gavage 4 mL twice daily for 7 d. Then, *AD*-containing serum (*AD*cs) was collected. MTS assay was applied to measure the VSMC viability. The proliferation of VSMCs was detected by 5-bromodeoxyuridine (BrdU) immunocytochemistry. The microRNA (miRNA) profiling was performed, and the target genes of miRNAs were searched from the TargetScan 7.2 database. The expressions of matrix metalloproteinases-2/9 (MMP-2/9), cyclin D1/E, cyclin-dependent kinase inhibitor 1B (p27), extracellular regulated protein kinases 1/2 (ERK1/2), and ERK1/2 phosphorylation were examined by western blotting or quantitative reverse transcription PCR. *Results*. The ox-LDL-induced miR-17-92a expression promoted VSMC proliferation. *AD* and the ERK1/2 inhibitor U0126 (10 *μ*mol/L) inhibited VSMC proliferation and reduced the overexpression of miR-17∼92a. *AD* was found to inhibit phosphorylation of ERK1/2 and reduced the expression of MMP-2/9 in VSMCs. The expression of cyclin D1/E was suppressed, and p27 was elevated following treatment with *AD* as well as ERK1/2 inhibitor. According to the TargetScan 7.2 database, the target genes of miR-17∼92a act on tissue inhibitors of metalloproteinases (TIMPs)-MMPs, p27/21 cyclins, and peroxisome-proliferator-activated receptor *α* (PPAR*α*) ATP-binding cassette transporter (ABC) A1/G1, which are involved in the process of atherosclerosis. *Conclusions*. *AD* inhibits ox-LDL-induced VSMC proliferation via inhibiting ERK1/2 and miR-17∼92a activation. The results provide the multitarget mechanisms for application of *AD* in the treatment of atherosclerosis. It would be helpful to the treatment of cardiovascular and cerebral diseases.

## 1. Introduction

As the main constitutive stromal cells of the vascular wall, vascular smooth muscle cells (VSMCs) are the only cell type in the medial layer of the arterial wall. It is recognized that VSMCs proliferate at a very low frequency in the normal vessel wall [1]. However, vascular injury has been found to induce aberrant VSMC proliferation and migration [2]. Increasing evidence shows that abnormal VSMC proliferation plays a central role in vascular pathogenesis [3, 4]. Cumulative evidence indicates that cell-cycle progression is mediated by cell-cycle regulatory proteins, such as cyclin D1, cyclin E, and cyclin-dependent kinase inhibitor 1B (p27), which are associated with G0/G1 phase cell-cycle arrest [5]. In vascular diseases, matrix metalloproteinases (MMPs) have been identified as a key enzyme in cell wall remodeling, plaque formation, and restenosis [6]. MMPs (MMP-2 and MMP-9) are reported to promote the proliferation of VSMCs [7] and repressed by the tissue inhibitors of metalloproteinases (TIMPs). In addition, MMP-2 and MMP-9 expression is stimulated by ox-LDL in VSMCs, which is mediated by the activation of the ERK signaling [8, 9].

A series of cellular behaviors, including differentiation, development, and proliferation, are under microRNA (miRNAs) control. miRNAs are small segments of noncoding sequences involved in the post-transcriptional regulation of gene expression [10]. It has been reported that miR-17∼92a clusters exert an important role on the proliferation of VSMCs, via targeting MMPs and nuclear factor kappa-B (NF-*κ*B) [11–13]. Interestingly, the miR-17∼92a cluster contains miR-17, miR-18a, miR-19a/b, miR-20a, and miR-92a, which are mediated by the extracellular regulated protein kinases 1/2 (ERK1/2)/ELK1 pathway in endothelial cells [14].

*Alismatis rhizoma* decoction (*AD*), a classic traditional Chinese medicinal formula used for the treatment of cardiovascular and cerebral diseases, consists of the combination of *Alisma orientalis* and *Atractylodes macrocephala* [15]. *AD* exhibited multiple pharmacological actions, including hepatoprotective, anti-inflammatory, anticancer, and antioxidant activities [9, 16–19]. Our previous study showed that *AD* reduced lipid deposition in macrophages-derived foam cells [20] and a triterpenoid derived from *Alisma orientale* (Alisol A 24-acetate) suppressed the migration and phenotypic transformation of ox-LDL-induced VSMCs [8]. However, little is known about the effect of *AD* on VSMC proliferation. The present study investigates the impact of *AD* on ox-LDL-induced VSMC proliferation and explores the underlying mechanisms.

## 2. Materials and Methods

### 2.1. Ethical Statement and Animals

This study was approved by the Ethics Review Committee of the Affiliated Rehabilitation Hospital of Fujian University of Traditional Chinese Medicine (FJKFYY-201400127). All animal experiments were performed in accordance with the Management of Animal Care and Use Programs in Research, Education, and Testing [21]. Male 6-week-old Sprague-Dawley (SD) rats, each weighing 200 to 220 g, were housed in a temperature-controlled (21°C) environment under a 12-h light/dark cycle and given free access to water and food.

### 2.2. Preparation of *Alismatis rhizoma* Decoction and *AD*cs

*AD* and *AD*cs was prepared as described previously [20]. Briefly, crude materials of *Alismatis rhizoma* decoction were supplied by the Fujian University of Traditional Chinese Medicine Rehabilitation Hospital Pharmacy and were carefully identified. *Alisma orientalis* and *Atractylodes macrocephala* (5 : 2) were soaked in water for 30 min and mixed in proportion and decocted twice by refluxing with water (1: 6 and then 1: 4, w/v) for 1 h. The filtrates were combined and condensed. They were then stored at 4°C until use. The SD rats were assigned to 2 groups (15 rats for each group). The *AD* group was given *AD* by oral gavage 4 mL twice daily for 7 d (approximately 2.56 g/mL *Alisma orientalis* and *Atractylodes macrocephala*; Affiliated Rehabilitation Hospital of Fujian University of Traditional Chinese Medicine, Fuzhou, China), and the control group was given 0.9% NaCl by oral gavage 4 mL twice daily for 7 d. Then, blood samples were collected from the rat abdominal aorta following induction of anesthesia with diazepam/ketamine (1 : 1) at a dose of 0.1 mL/100 g, and serum was separated by centrifugation at 3000 rpm at 4°C for 10 min and inactivated in a 56°C water bath for 30 min. Then, the bacteria were filtered through a microporous membrane, and the serum was stored at −20°C for the subsequent experiments.

### 2.3. Isolation and In Vitro Culture of Rat VSMCs and Grouping

Rats were sacrificed, and the thoracic aorta was isolated under aseptic conditions as described previously [8]. The intima and adventitia of the thoracic aorta were removed, and the specimens of thoracic aorta were cut into pieces measuring 1 × 1 mm, digested with type II collagenase at 37°C for 14 h, and then incubated in the DMEM/F-12 medium (Hyclone, Logan, UT, USA) supplemented with 20% fetal bovine serum (FBS; Hyclone). The culture medium was replaced once every 2-3 days with the DMEM medium supplemented with 10% FBS at 37°C in humidified atmosphere containing 5% CO_2_. VSMCs from 4 to 8 passages were used for experiments. Cells grown to 80–95% confluence were made quiescent by serum starvation for 24 h, and then VSMCs were grouped. Cells in the ox-LDL group were cultured in the DMEM/F12 medium supplemented with 20% normal rat serum containing 50 mg/L ox-LDL (Peking Union-Biology Co, Ltd., Beijing, China) for 12, 24, and 48 h, cells in the *AD* group were cultured in the DMEM/F12 medium supplemented with 20% *AD*cs and 50 mg/L ox-LDL for 12, 24, and 48 h, and cells in the U0126 group were cultured in the DMEM/F12 medium supplemented with 20% rat serum containing 10 *μ*mol/L U0126 (Sigma-Aldrich, Inc., St. Louis, MO, USA) and 50 mg/L ox-LDL for 12, 24, and 48 h, while cells in the control group were cultured in the DMEM/F12 medium supplemented with 20% normal rat serum for 12, 24, and 48 h.

### 2.4. MTS Assay

To measure the cell viability, VSMCs were seeded onto 96-well plates (Corning, Inc.; Corning, NY, USA) at a density of 1 × 10^4^ cells per well and cultured for 24 h until 80% confluence. Following incubation in serum-free medium during the initial 24 h, VSMCs were exposed to *AD*cs at concentrations of 0%, 10%, 20%, 30%, 40%, and 50% for 12 and 24 h, respectively. Then, the viability of VSMCs was measured using the MTS assay (Promega, Madison, WI, USA) following the manufacturer's instructions.

### 2.5. Incorporated BrdU of Immunohistochemistry Assay for VSMC Proliferation

To measure the VSMC proliferation, VSMCs were seeded onto 6-well plates at a density of 5 × 10^5^ cells per well and incubated in the DMEM medium supplemented with 10% FBS for 24 h, followed by serum starvation for 24 h to make a synchronization of cell growth in the G0 phase of the cell cycle. They were then assigned to 4 groups: the control group, ox-LDL group, ox-LDL + 20% *AD*cs, and ox-LDL + U0126 group. Following treatment with ox-LDL, 20% *AD*cs, and U0126 for 12 and 24 h, BrdU (30 *μ*mol/L; APExBIO, USA) was added to the cell culture medium 12 h before collecting the cells. Immunocytochemical staining for BrdU expression was performed on VSMCs by the SP method and following the manufacturer's instructions. Briefly, VSMCs were fixed in 4% paraformaldehyde, rinsed with PBS, 2 mol/L hydrochloric acid for 30 min at 37°C, blocked with goat serum, incubated with mouse anti-rat BrdU monoclonal antibody (1 : 1000; Proteintech, CA, USA), rinsed with PBS, incubated with biotin-conjugated goat anti-mouse IgG antibody (1 : 1000; BOSTER Biological Technology, Wuhan, China), followed by incubation in streptavidin, avidin, and peroxidase, visualized with diaminobenzidine (DAB; Maxim-Bio, Fuzhou, China) and DAPI dye solution (100 ng/mL; BOSTER Biological Technology, Wuhan, China), drained the dye solution, and observed under microscopy. In the negative control group, the primary antibody was replaced with PBS. Five fields of vision were randomly selected from each slip under a microscope at 400× magnification and then photographed. The images captured were analyzed using the image processing software Image-Pro Plus v6.0 (Media Cybernetics, Bethesda, MD, USA). Positive BrdU expression was defined as the presence of pale-yellow or deep-tan staining, while nonspecific background staining was excluded. Positive DAPI cells were defined as the presence of blue. All experiments were repeated in triplicate. The ratio of BrdU-positive cells and total cells was estimated.

### 2.6. miRNA Microarray Assay

The cells were treated as described above. Total RNA was extracted from VSMCs. The miRNA microarray profiling was performed using the Lianchuan Biological Small RNA Sequencing Analysis (Lianchuan Bio, China) according to the manufacturer's protocol. Small RNA sequencing library preparation was performed using the TruSeq Small RNA Sample Prep Kits (Illumina, San Diego, CA USA) kit. After the library preparation work was completed, the constructed library was sequenced using Illumina Hiseq 2000/2500, and the sequencing read length was single-ended 1 × 50 bp.

### 2.7. Western Blotting Analysis

Protein was extracted from VSMCs, and the protein concentration was quantified with the BCA protein assay kit (TransGen Biotech Co., Ltd., Beijing China). Total protein was separated by 10% SDS-PAGE, and the blots were electrotransferred to a PVDF membrane with a 0.45 *μ*m pore size (Millipore, Billerica, MA, USA). The membranes were blocked with 5% (*w*/*v*) fat-free milk in 0.1% (*v*/*v*) TBST at room temperature for 2 h. Then, the immunoblots were incubated with the primary anti-MMP-2 (1 : 1000; Cell Signaling Technology, Inc., Danvers, MA, USA), anti-MMP-9 (Abcam, Cambridge, UK), anti-p27^Kip1^ (Abcam, Cambridge, UK), anti-Cyclin E (1 : 1000; Cell Signaling Technology, Inc., Danvers, MA, USA), anti-Cyclin D1 (1 : 1000; Cell Signaling Technology, Inc., Danvers, MA, USA), anti-p-Erk1/2 (1 : 1000; Cell Signaling Technology, Inc., Danvers, MA, USA), and anti-Erk1/2 (1 : 1000; Cell Signaling Technology, Inc., Danvers, MA, USA) at 4°C overnight, while anti-*β*-actin mouse monoclonal antibody (BOSTER Biological Technology Co, Ltd., Wuhan, China) served as a loading control. The membranes were then washed three times with TBST, of 10 min each time, and incubated with anti-mouse or anti-rabbit IgG HRP-conjugated secondary antibodies (1 : 3000; BOSTER Biological Technology Co, Ltd., Wuhan, China) at room temperature for 1 h. After the membranes were rinsed three times with the wash buffer, the peroxidase activity was detected by an enhanced chemiluminescence (ECL) kit with the ChemiDoc XRS^+^ system (Bio-Rad Laboratories, Inc., Hercules, CA, USA), and then the quantification of the band intensity was analyzed using the Image Lab Software, version 3.0 (Bio-Rad Laboratories, Inc., Hercules, CA, USA).

### 2.8. qRT-PCR Assay

Total RNA was extracted from VSMCs with the Trizol reagent (TaKaRa Biotechnology, Dalian, China) following the manufacturer's instructions, and the RNA concentration was quantified. The total RNA was reversely transcribed into cDNA. The expression of MMP-2 and MMP-9 was quantified using the qRT-PCR assay according to the previous study [19]. PCR was performed according to the manufacturer's instructions. According to GenBank, the RT-PCR primers were designed as follows: MMP-9 forward: 5′-CAAGGACGGTCGGTATTGGAAG-3′ and reverse: 5′-AAACGAGTAACGCTCTGGGGAT-3′, with 348 bp in length; MMP-2 forward: 5′-TCCCGTTATGAGACCCTGAGC-3′ and reverse: 5′-AGGACGCA- GAGAACCCTGAGAG-3′, with 205 bp in length; and GAPDH forward: 5′-ACGGCAAGTTCAACGGCACAG-3′ and reverse: 5′-GAAGACGCCAGTAGACT CCACGAC-3′, with 149 bp in length. The PCR products were checked by electrophoresis on 1.5% agarose gel, and the data were analyzed using the Image Lab Software, version 3.0. The relative mRNA expression level was estimated by the ratio of the gray scale value of target gene to GAPDH.

### 2.9. Statistical Analysis

All the experimental data were expressed as mean ± SD. All statistical analyses were performed using the statistical software SPSS, version 18.0 (SPSS, Inc., Chicago, IL, USA), of three independent experiments and assessed by one-way analysis of variance (ANOVA) followed by Fisher's protected least-significant difference test to compare two treatments, and *P* value <0.05 was considered statistically significant.

## 3. Results

### 3.1. Morphology of Rat VSMCs

The rat VSMCs appeared as a single shape or an irregular triangle, with abundant high-intensity cytoplasm, and the nucleus appeared round and was located in the center. After growing to confluence, VSMCs displayed a typical peak and valley growth morphology (Supplementary Materials available here). As shown in Supplementary Materials, cells were positive for *α*-smooth muscle actin (*α*-SMA, 1 : 1000; BOSTER Biological Technology, Wuhan, China) staining and myofilaments in the cytoplasm ran along the long axis of the cell. Therefore, cells were identified as VSMCs.

### 3.2. Cytotoxicity of *AD*cs to VSMCs

**Figure 1:**
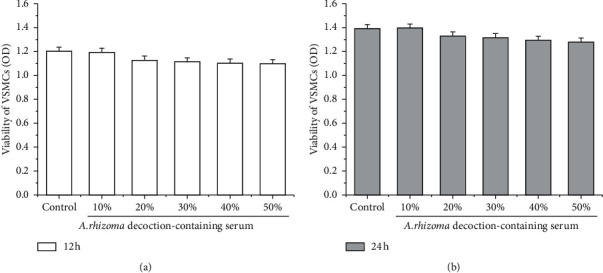
Effects of *AD* on the viability of VSMCs. Cells are exposed to *AD* at concentrations of 10%, 20%, 30%, 40%, and 50% for 12 h (a) and 24 h (b), respectively. MTS assay shows 20% *AD* had no cytotoxicity to VSMCs.

There was no significant difference in body weight, mortality, and disability between the *AD* group and the control group during the preparation of *AD*cs. Quiescent VSMCs were exposed to *AD*cs at concentrations of 10%, 20%, 30%, 40%, and 50% for 12 (Figure 1(a)) and 24 h (Figure 1(b)) in the absence of ox-LDL, and the MTS assay revealed no significant difference in the viability of VSMCs treated with *AD*cs as compared with untreated cells. The results indicated that *AD*cs had no cytotoxicity to VSMCs, and 20% *AD*cs was selected as an intervention concentration.

### 3.3. *AD* Inhibits ox-LDL-Induced VSMC Proliferation

**Figure 2:**
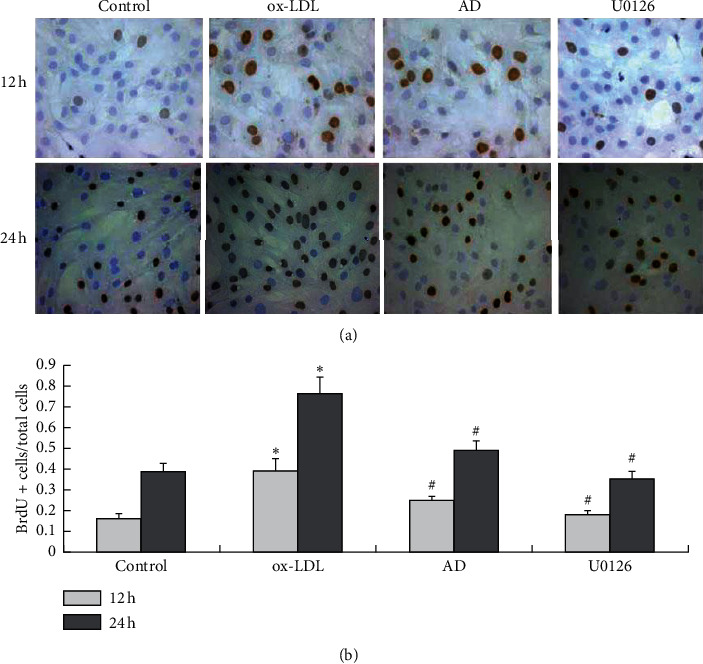
*AD* inhibits ox-LDL-induced VSMC proliferation. Following treatment with ox-LDL (50 mg/L), *AD*, and the ERK1/2 inhibitor U0126 (10 *μ*mol/L) for 12 and 24 h, BrdU-positive cells and total cells were calculated. These data are representative of 3 experiments. Results are described as the mean ± SD (*n* = 3). ox-LDL vs. control group (^*∗*^*p* < 0.01); *AD* vs. ox-LDL group (^#^*p* < 0.01), and U0126 vs. ox-LDL group (^#^*p* < 0.01).

Incorporated BrdU of immunocytochemistry assay revealed that ox-LDL treatment increased the number of BrdU-positive cells (new proliferation of VSMCs) (*p* < 0.01, Figure 2); however, treatment with *AD* markedly reduced the number of BrdU-positive VSMCs at 12 and 24 h (*p* < 0.01, Figure 2), indicating that *AD* may suppress ox-LDL-induced VSMC proliferation.

### 3.4. *AD* Inhibits ERK1/2 Phosphorylation in ox-LDL-Induced VSMCs

**Figure 3:**
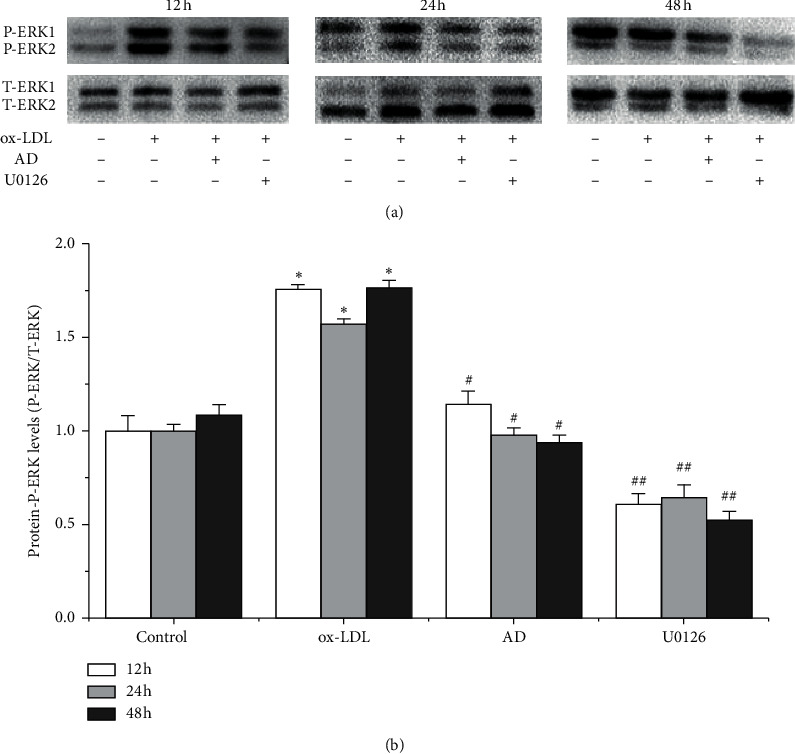
Inhibition of *AD* on phosphorylation of ERK1/2 in ox-LDL-induced VSMCs. Whole cell lysates were prepared and subsequently used for detection of p-ERK1/2 and ERK1/2 expressions by Western blotting. ^*∗*^*p* < 0.05 ox-LDL vs. control group; ^#^*p* < 0.05*AD* vs. ox-LDL group; ^##^*p* < 0.01 U0126 vs. ox-LDL group.

Since the ERK1/2 signaling pathway is associated with the proliferation of VSMCs, we assessed the effect of *AD* on the activation of the ERK1/2 pathway. Western blotting assay showed ox-LDL increased the expression of p-ERK1/2 in VSMCs (*p* < 0.05, Figure 3), and the treatment with *AD* and U0126 significantly suppressed p-ERK1/2 expression at 12, 24, and 48 h (*p* < 0.05 for *AD* and *p* < 0.01 for U0126, Figure 3). These results suggested that *AD* inhibited ox-LDL-induced VSMC proliferation through downregulating the activity of the ERK1/2 signaling pathway.

### 3.5. *AD* Regulates miR-17∼92a Cluster Expression in ox-LDL-Induced VSMCs via ERK1/2 Pathway

**Figure 4:**
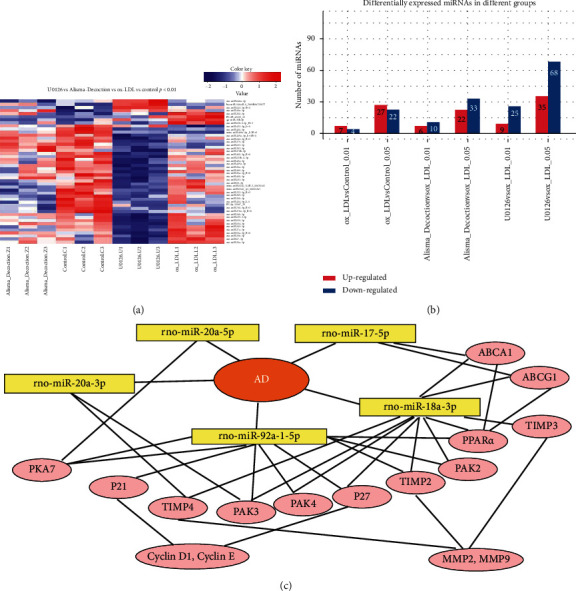
miRNA microarray assay shows that AD and the ERK1/2 inhibitor regulates different miRNA expressions in ox-LDL-induced VSMCs. AD and the ERK1/2 inhibitor U0126 (10 *μ*mol/L) suppressed miR-17∼92a cluster overexpression in ox-LDL-induced VSMCs. Target genes of mir-17∼92a cluster are related to TIMPs-MMPs and cell-cycle regulatory proteins according to the TargetScan7.2 database. TIMP-2, TIMP-3, and TIMP-4 are regulated by miR-20a-3p, miR-92a-1-5p, and miR-18a-3p. p21 and p27 were regulated by miR-92a-1-5p and miR-18a-3p, and the miR-17∼92a cluster acts on the p21 activated kinases (PAks) according to TargetScan7.2 database prediction.

To determine whether miRNAs were involved in the effect of *AD* on ox-LDL-induced VSMC proliferation, miRNA microarray analysis was used to detect the miRNA expression in ox-LDL-induced VSMCs. The miRNA microarray assay showed that there were lots of different miRNA expressions among four groups (Figures 4(a), 4(b)). Furthermore, we found that ox-LDL upregulated the levels of the miR-17∼92a cluster (miR-18a-3p, miR-20a-5p, miR-92a-3p) in VSMCs (*p* < 0.05, Table 1). miR-17-5p, miR-19b-1-5p, miR-20a-3p, and miR-92a-1-5p in VSMCs were upregulated by ox-LDL, but it was not significant (*p* < 0.1). *AD* dramatically reduced the expression of miR-17∼92a cluster in ox-LDL-induced VSMCs (*p* < 0.01 for miR-17-5p and miR-20a-3p, *p* < 0.05 for miR-18a-3p, miR-20a-5p, and miR-92a-1-5p, Table 1). *AD* also inhibited the miR-19b-1-5p (*p* = 0.08) and miR-92a-3p (*p* = 0.10) expression in ox-LDL-induced VSMCs, but it was not a significant difference (*p* > 0.05). Meanwhile, we found that blocking the ERK1/2 pathway completely suppressed the miR-17∼92a cluster overexpression in the ox-LDL-induced VSMCs (*p* < 0.01 for miR-17-5p and miR-20a-5p, *p* < 0.05 for miR-18a-3p, miR-19b-1-5p, miR-20a-3p, miR-92a-3p, and miR-92a-1-5p, Table 1). The sequences of miR-17∼92a cluster are showed in Table 2. The data indicted that *AD* inhibited the overexpression of miR-17∼92a cluster and ERK1/2 pathway activation in the ox-LDL-induced VSMCs.

Exploring the role of miR-17∼92a cluster in the VSMC proliferation, we searched for its possible target genes according to the TargetScan 7.2 database. Target genes of miR-17∼92a cluster are directly related to the expression of TIMP-2, TIMP-3, TIMP-4, p21, p21-activated kinases (PAks), and p27, which are associated with cell proliferation (Figure 4). TIMP-2, TIMP-3, and TIMP-4 are the important factors for the expression of MMP-2 and MMP-9. p21 and p27 show the negative correlation with cyclin D1 and cyclin E expressions, which are the target genes of miR-92a-1-5p and miR-18a-3p in the miR database.

### 3.6. *AD* Suppresses ox-LDL-Induced Upregulation of MMP-2 and MMP-9 Expressions in VSMCs and U0126

**Figure 5:**
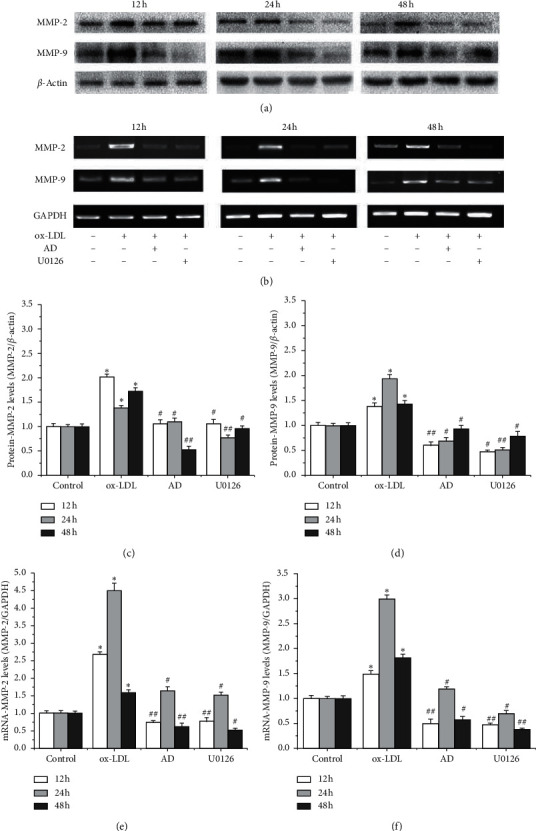
*AD* and the ERK 1/2 blocker inhibit the expression of MMP-2 and MMP-9 in ox-LDL-induced VSMCs. Western blot analysis and qPCR assay were performed to determine the expression of MMP-2 and MMP-9, while *β*-actin and GAPDH serves as a loading control. The graphs represent the relative expression of these proteins and mRNAs for three independent experiments. ^*∗*^*p* < 0.05 ox-LDL vs. control group; ^#^*p* < 0.05*AD* or U0126 vs. ox-LDL group; ^##^*p* < 0.01*AD* or U0126 vs. ox-LDL group.

MMP-2 and MMP-9 protein expressions significantly increased following the ox-LDL treatment (*p* < 0.05 for 12, 24, and 48 h), MMP-2 protein upregulation was inhibited by *AD* (*p* < 0.05 for 12 and 24 h, *p* < 0.01 for 48 h), and MMP-9 protein upregulation was suppressed by *AD* (*p* < 0.01 for 12 h, *p* < 0.05 for 24 h and 48 h, Figure 5). The overexpression mRNAs of MMP-2 and MMP-9 in ox-LDL-induced VSMCs were inhibited by *AD* (*p* < 0.05, Figure 5). Similar results were found in the ERK1/2 blocker U0126 treatment.

### 3.7. Effects of *AD* and ERK1/2 Blocker U0126 on Cell-Cycle Regulatory Proteins in VSMCs

**Figure 6:**
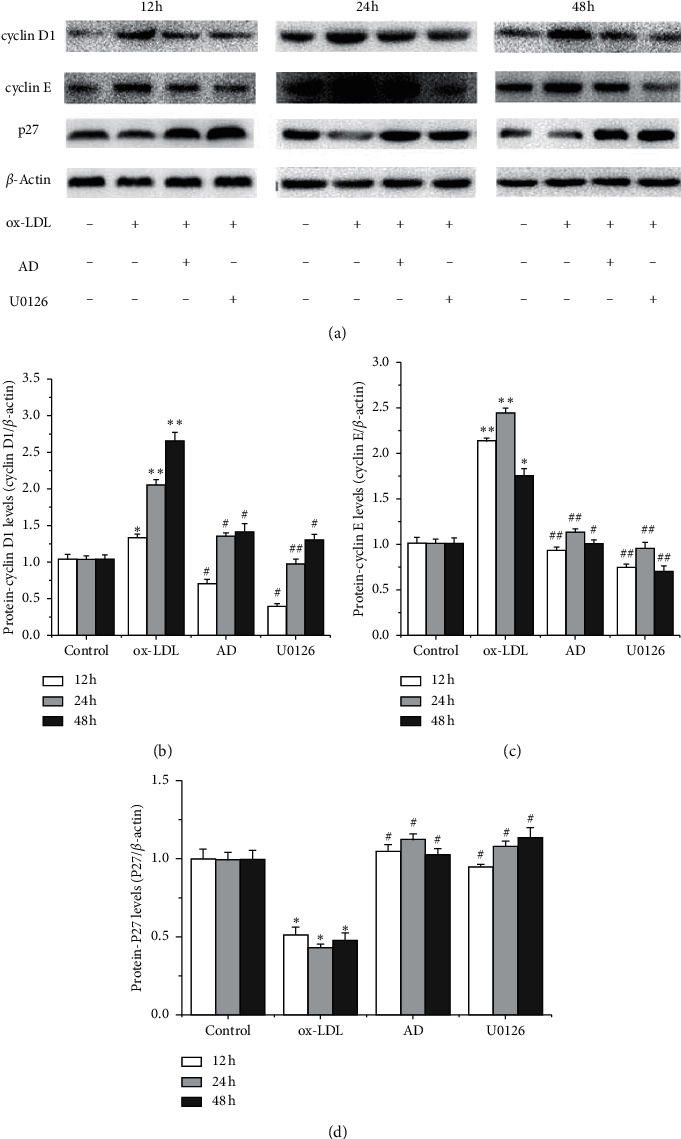
Effects of *AD* and ERK1/2 inhibitor U0126 on the expression of cell-cycle regulatory proteins in ox-LDL-induced VSMCs. Western blot analysis was performed to determine the expression of cyclin D1, cyclin E, and p27, while *β*-actin served as a loading control. The graphs represented the relative expression of these proteins for three independent experiments. ^*∗*^*p* < 0.05 ox-LDL vs. control group; ^*∗*^*p* < 0.01 ox-LDL vs. control group; ^#^*p* < 0.05*AD* or U0126 vs. the ox-LDL group; ^##^*p* < 0.01*AD* or U0126 vs. the ox-LDL group.

As *AD* inhibited ox-LDL-induced VSMC proliferation, we hypothesized that *AD* may be associated with the expression of cell-cycle-associated proteins in VSMCs. Western blotting showed that ox-LDL increased expression of cyclin D1 and cyclin E in VSMCs (*p* < 0.05 for 12 h, *p* < 0.01 for 24 and 48 h) and decreased the p27 expression; *AD* and U0126 dramatically inhibited the overexpression of cyclin D1 and cyclin E (*p* < 0.01 for 12, 24, and 48 h). However, the expression of p27 was upregulated following the exposure to *AD* and U0126 (*p* < 0.05 for 12, 24, and 48 h, Figure 6).

## 4. Discussion

As an ancient classical traditional Chinese medicinal formula, *AD* (*Alisma orientalis* and *Atractylodes macrocephala*), was first described in the Eastern Han Dynasty and has been used for the treatment of cardiovascular and cerebral diseases in China for decades [16, 17, 20]. AD with the ED50 value of 8.04 g/mL and IC50 value of 3.15 or 5.14 g/mL were showed in cellular experiments [22, 23]. The rats treated with AD (2.56 g/mL, 4 mL twice daily) were proved to be effective, and no side effects in our study were found. During atherogenesis, the proliferation and migration of arterial VSMCs from media to intima had been shown to contribute to neointimal VSMC accumulation [4], where ox-LDL serves as a chemoattractant to VSMCs [24]. Although *AD* exhibits a wide range of bioactivities in diverse cells [8, 20], its effect on ox-LDL-induced proliferation of VSMCs remain unknown until now. The present *in vitro* study was therefore designed to examine the potential role of *AD* through suppressing the proliferation of VSMCs stimulated by ox-LDL.

It has been proposed that the differentiation, proliferation, and apoptosis of cells are regulated by miRNAs. More than one-third of protein-coding genes are under miRNA translational control, with a single miRNA able to regulate hundreds of protein-coding genes. An miR-17∼92a cluster is a group of highly conserved miRNAs, located at chromosome 13q31-q32 [25], including miR-17, miR-18a, miR-19a/b, miR-20a, and miR-92a; miR-17∼92 is required for normal animal development and cell proliferation, individual member of the miR-17∼92 cluster appears to possess distinct function [26–30]. Several studies reported that miR-17∼92 played a key role in the regulation of cardiomyocyte proliferation [26] and miR-17 stimulated the proliferation of VSMCs, enhanced cell-cycle G1/S transition [11], and regulated p21 expression [30]. In the present study, we found that ox-LDL upregulated miR-17∼92a cluster expression and increased the number of new proliferations of VSMCs, *AD* treatment resulted in a significant reduction of miR-17∼92a expression, and the capacity of proliferation in ox-LDL-induced VSMCs was markedly inhibited following the treatment with *AD*. It has been recently confirmed that the miR-17∼92a cluster are mediated by the ERK pathway in endothelial cells [16] and miR-18a promotes cell proliferation via AKT and ERK pathways [31]. We found ox-LDL-induced the phosphorylation of ERK1/2 in VSMCs, *AD* inhibited the ERK1/2 pathway activation, and blocking ERK1/2 pathways dramatically suppressed miR-17∼92a cluster expression. We consider that *AD* may inhibit ox-LDL-induced VSMC proliferation, which is associated with the ERK1/2 pathway and miR-17∼92a cluster activation.

VSMC proliferation has shown an essential role in the progression of atherosclerotic lesions. p21 and p27 are regulated by miR-92a-1-5p and miR-18a-3p, and the miR-17∼92a cluster acts on p21-activated kinases (PAks) according to the TargetScan7.2 database prediction. As a negative regulator of cyclins, p27, p21, and PAks are reported to play an important role in the cell-cycle arrest [5, 32]. It is known that cyclin D, cyclin E, and p27 are important cyclins in cell-cycle progression, which are closely related to cell proliferation [33]. Progression through the G1 phase of the cell cycle is known to be controlled by cyclin D1 and cyclin E, and it is reported that cyclin D1 and cyclin E are necessary for transition from G1 to S phase, which is also known as the DNA synthesis phase. Physiologically, p27 maintains at a high level in the G0/G1 phases and allows cells at the G1 phase and quiescent [32]. Upon mitogenic stimulation, p27 is rapidly degraded, thus enabling cyclin D1 and cyclin E to promote cell proliferation. We detected that treatment with *AD* and ERK1/2 inhibitor U0126 increased p27 protein expression and reduced the expression of cyclin D1 and cyclin E proteins in VSMCs exposed to ox-LDL, suggesting that treatment with *AD* and ERK1/2 blocker upregulated p27 expression and inhibited cyclin D1 and cyclin E expressions, which may be linked to miR-17∼92a cluster expression.

It was estimated that MMP-2 and MMP-9 in VSMCs were crucial in the vascular remodeling [34] and the constitutive expression of MMP-2 and MMP-9 has been linked to VSMC proliferation [35, 36]. Inhibition of MMP-2 and MMP-9 has been employed as a potential strategy to prevent atherosclerosis. In this study, *AD* suppressed the overexpression of MMP-2 and MMP-9 in ox-LDL-induced VSMCs. Similar effects were found in ox-LDL-treated VSMCs exposed to the ERK1/2 inhibitor U0126. Xu reported that the overexpression of miR-20a suppressed TIMP-2 expression and increased the expression of MMP-2 and MMP-9 [37]. It has been reported that the miR-92a expression in VSMCs is low in the quiescent state and miR-92a regulates the expression levels of MMP-9 and TIMP-3 in the hydrogen-peroxide-induced VSMCs [13]. According to the TargetScan7.2 database, TIMP-2, TIMP-3, and TIMP-4 are the target genes of miR-20a-3p, miR-92a-1-5p, and miR-18a-3p, and TIMPs is the negative regulator of MMPs. The data showed that *AD* regulated the miR-17∼92a cluster expression and further may affect the TIMP-MMP expression in the ox-LDL-induced VSMCs.

Several targets of miR-17∼92a in the process of atherosclerosis have been predicted. According to the TargetScan7.2 database, mir-17a-5p and mir-18a are associated with ATP-binding cassette transporter A1 (ABCA1) and ATP-binding cassette transporter G1 (ABCG1), which are the important factors in atherosclerosis. It has been demonstrated that mir-17a-5p is associated with ABCA1 expression [23, 38] and lipid metabolism. Upregulation of ABC protein family (ABCA1 and ABCG1) can enhance the cholesterol efflux and reduce the formation of aortic plaques [39]. PPAR-alpha and PPAR-gamma activators induce the expression of ABCA1, which controls apoAI-mediated cholesterol efflux from macrophages [40]. PPAR-alpha is a major regulator of lipid metabolism and regulated by miR-92a-1-5p and miR-18a-3p. Therefore, *AD* may regulate atherosclerosis associated with the lipid regulation of mir-17∼92a cluster.

## 5. Conclusion

The results of our study demonstrate that treatment with *AD* inhibits ox-LDL-stimulated VSMC proliferation, which is strongly associated with the inhibition of the ERK1/2 and miR-17∼92a activation. Our data provide new insights into the mechanisms of *AD* on atherosclerosis, and it provides a theoretical basis for the clinical application of *AD.*

## Figures and Tables

**Table 1:** Individual member of miR-17∼92a cluster expression in the groups.

mir17∼92a cluster	Control	ox-LDL	AD	U0126
miR-17-5p	2886 ± 379.16	3619 ± 201.26	2815 ± 355.45^*∗∗*^	1859 ± 269.48^ΦΦ^
miR-18a-3p	14.05 ± 3.594	21.88 ± 2.354^#^	14.67 ± 3.786^*∗*^	10.2685 ± 4.931^Φ^
miR-19b-1-5p	2.667 ± 2.11	5.37 ± 2.37	1.53 ± 0.800^Δ^	1.6867 ± 1.0781^Φ^
miR-20a-3p	12.01 ± 6.735	17.50 ± 2.03	12 ± 0.378^*∗∗*^	11 ± 1.187^Φ^
miR-20a-5p	5671 ± 1404.02	7837 ± 184.636^#^	6428 ± 804.8586^*∗*^	3669 ± 723.56^ΦΦ^
miR-92a-3p	4893 ± 169.03	6182 ± 306.56^#^	5503 ± 266.32	3326 ± 197.84^Φ^
miR-92a-1-5p	2.05 ± 1.21	3.34 ± 0.84	1.33 ± 1.11^*∗*^	0.78 ± 0.73^Φ^

^#^ *p* < 0.05 ox-LDL vs. control; ^*∗*^*p* < 0.05, ^*∗∗*^*p* < 0.05 ADcs vs. ox-LDL; ^Δ^*p*=0.08 ADcs vs. ox-LDL; ^Φ^*p* < 0.05; ^ΦΦ^*p* < 0.01 U0126 vs. ox-LDL.

**Table 2:** The sequences of individual member in miR-17∼92a cluster.

miR-17-5p	CAAAGTGCTTACAGTGCAGGTAG
miR-18a-3p	ACTGCCCTAAGTGCTCCTTCTGA
miR-19b-1-5p	AGTTTTGCAGGTTTGCATCCAGC
miR-20a-3p	ACTGCATTACGAGCACTTACAG
miR-20a-5p	TAAAGTGCTTATAGTGCAGGTAG
miR-92a-3p	TATTGCACTTGTCCCGGCCTGT
miR-92a-1-5p	AGGTTGGGATTTGTCGCAATGCT

## Data Availability

The data sets used and/or analyzed during the current study are available from the corresponding author on reasonable request.

## Supplementary Materials

Supplementary Materials Supplementary figure: morphology of primary cultured rat VSMCs in vitro. A. VSMCs grow to form a monolayer, which displays a spindle-like pattern. B. Immunofluorescent test for *α*-SMA expression in VSMCs.
