# Supplementary figures and images for: Proliferation of Vascular Smooth Muscle Cells under ox-LDL Is Regulated by Alismatis rhizoma Decoction via InhibitingERK1/2 and miR-17∼92a Cluster Activation
Source: Evid Based Complement Alternat Med. 2020 Aug 22;2020:7275246. doi: 10.1155/2020/7275246 (PMC7463403; doi:10.1155/2020/7275246)

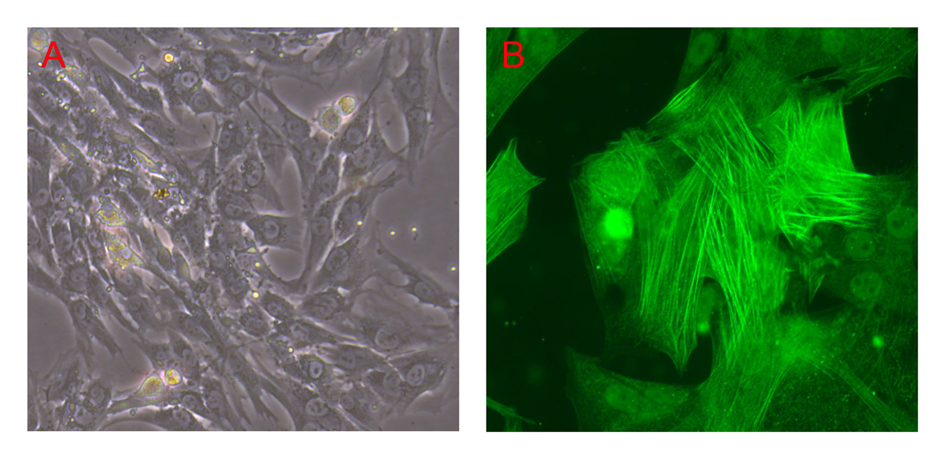

Supplement: Supplementary Materials — Supplementary figure: morphology of primary cultured rat VSMCs in vitro. A. VSMCs grow to form a monolayer, which displays a spindle-like pattern. B. Immunofluorescent test for α-SMA expression in VSMCs. [file 7275246.f1.tif]
